# Whole-body Dynamic [^18^F]FDG-PET/CT in Giant Cell Arteritis and Polymyalgia Rheumatica

**DOI:** 10.1097/RLU.0000000000006369

**Published:** 2026-03-10

**Authors:** Bert-Ram Sah, Alexey Eyrikh, Lars Husmann, Aresh Farokhnia, Urs J. Muehlematter, Stephan Beintner-Skawran, Fotis Kotasidis, Munenobu Nogami, Junko Inoue Inukai, Alexander Maurer, Martin W. Huellner

**Affiliations:** * Department of Nuclear Medicine, University Hospital Zurich, University of Zurich, Zurich, Switzerland; † Department of Diagnostic, Interventional, and Pediatric Radiology, Inselspital, University of Bern, Bern, Switzerland; ‡ Department of Immunology, University Hospital Zurich and University of Zurich, Zurich, Switzerland; § GE HealthCare, Waukesha, WI; ∥ Department of Radiology, Kobe University Graduate School of Medicine, Kobe, Japan; ¶ Division of Medical Imaging, Biomedical Imaging Research Center, University of Fukui, Fukui, Japan

**Keywords:** GCA, polymyalgia rheumatica, dynamic PET, Patlak, inflammation, vasculitis

## Abstract

**Objectives:**

We aimed to explore the diagnostic efficacy of whole-body dynamic (WBD) acquisition using [^18^F]-Fluorodeoxyglucose-positron-emission-tomography/computed-tomography ([^18^F]FDG-PET/CT) for the assessment of giant cell arteritis (GCA) and polymyalgia rheumatica (PMR) compared with the standard static PET/CT.

**Methods:**

Twenty-five patients with suspected GCA and/or PMR were prospectively enrolled in this single-center study. WBD PET imaging was performed before treatment using a standard PET/CT scanner with a multibed multipass dynamic whole-body acquisition approach (dPET). Reconstructed datasets were used to generate metabolic rate of [^18^F]FDG (MR_FDG_) images based on standard Patlak analysis. In case of pathologic FDG uptake, VOIs placed in vessel walls and joints were used for quantitation and definition of the target-to-background ratio (TBR): uptake in VOIs of vessel walls and joints (maximum values), to VOIs in blood pool (GCA), or to VOIs in normal muscles (PMR) (mean values). The final diagnosis based on the available imaging and laboratory results (except dPET), served as the standard of reference.

**Results:**

Seventeen of the 25 patients (68%) showed pathologic FDG uptake, of which 5 (20%) were finally diagnosed with GCA alone, 6 (24%) with PMR alone, and another 6 (24%) with both conditions. TBR difference between dPET and static PET was significant in both diseases(GCA *P*<0.003, PMR *P*<0.003), and TBRs of dPET and static PET were significantly higher in both diseases compared with the healthy controls.

**Conclusions:**

WBD [^18^F]FDG-PET/CT provided a significantly higher TBR in patients with GCA and PMR compared with standard static PET imaging. WBD [^18^F]FDG-PET/CT may potentially enhance the diagnostic accuracy in detecting GCA and PMR.

Giant cell arteritis (GCA) and polymyalgia rheumatica (PMR) are autoimmune diseases characterized by inflammation of large blood vessels and musculoskeletal structures, and they often coexist. There is an annual incidence of 10-50 individuals per 100,000 people per year worldwide, with an increasing global prevalence.^1,2^

Patients with vasculitis may present with nonspecific symptoms, such as fever, but more specific manifestations, including arm claudication and carotidynia, may also occur.^3^ Across all forms of vasculitis, laboratory tests typically reveal elevated levels of inflammatory markers. PMR is a rheumatic inflammatory condition that affects the bursae, tendons, tendon sheaths, and joints.^3–6^ With advancements in imaging, the overlap between these 2 diseases within the spectrum of rheumatic inflammatory conditions has become increasingly evident.^7,8^ Early and accurate diagnosis is essential to guide treatment strategies and prevent severe complications.^9^

Static [^18^F]FDG-PET/CT is an established imaging modality in the diagnosis and management of GCA and PMR.^7,10–18^ It provides a comprehensive, noninvasive assessment of inflammation, enabling visualization of increased metabolic activity in affected arteries, joint capsules, and tendons, offering more than just morphologic insights into these structures.^19,20^ [^18^F]FDG-PET/CT is particularly valuable for assessing patients with clinical symptoms, as it reveals the extent of vessel involvement, overall disease activity, and coexistence of PMR.

Studies indicate that the standard 60-min uptake time may be insufficient for optimal visualization of FDG accumulation in inflamed vessel walls.^7^ Intracellular FDG uptake in activated macrophages within the vessel wall is a progressive advancement that often does not reach its peak within this period. In addition, substantial residual activity in the blood pool at 60 min may obscure vessel wall inflammation. Delayed imaging, beyond 60 min, can result in a higher TBR.^20–22^ Research suggests that extending the uptake time to 2-3 hours may be more effective for detecting activity in vessel walls.^11,23,24^ However, prolonging the uptake time poses a practical challenge. A longer waiting period increases patient discomfort and resource utilization, as uptake rooms remain occupied for extended periods. In addition, the radioactive decay of the tracer necessitates higher initial doses to maintain the image quality, which could lead to a higher overall radiation dose. Balancing the benefits of delayed imaging with these logistical and patient-related considerations is critical for clinical practice.

Recent efforts have explored the potential of dynamic PET imaging as an alternative to static or extended static imaging protocols. Dynamic PET, particularly utilizing Patlak graphical analysis, allows for the differentiation between metabolized FDG in tissues and unmetabolized FDG in tissues and within the blood pool.^25^ Preliminary studies have indicated that whole-body dynamic PET may surpass static imaging in its ability to distinguish between benign and malignant findings.^26–28^

Our study aimed to investigate the diagnostic efficacy of whole-body dynamic [^18^F]FDG-PET/CT in comparison with standard static PET in assessing patients with GCA and PMR.

## PATIENTS AND METHODS

### Patients and Data Collection

**FIGURE 1:**
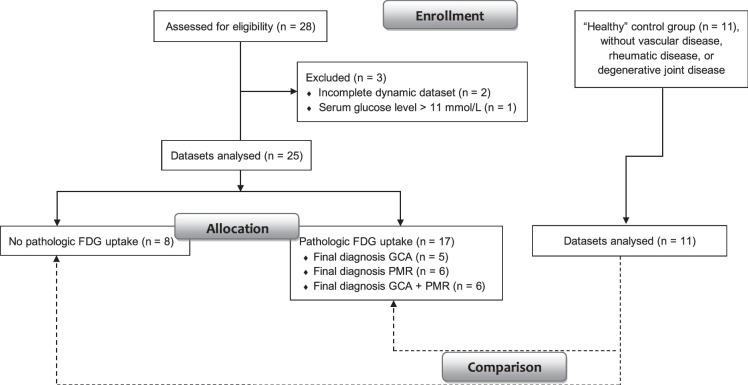
Patient flow chart. Flow chart showing patient selection and inclusion/exclusion through the study, with the number of participants at each stage.

Following Institutional Ethics Committee approval and obtaining individual informed consent, a total of 25 consecutive patients with suspected GCA or PMR were prospectively enrolled in this single-center study. The patients were recruited between January 2021 and July 2024. The control group consisted of 11 consecutive nononcologic subjects who did not suffer from vascular disease, rheumatic disease, or degenerative joint disease, and is defined as “healthy” in the context of our study (see below), and who underwent dynamic PET for another study (Fig. 1). Final diagnoses were made by a multidisciplinary clinical board, considering data on demographics, symptoms, physical signs, laboratory tests, biopsy results (in case of GCA), standard static PET imaging, and/or results of other imaging modalities, for example, ultrasound or magnetic resonance imaging (MRI). The clinical experts considered the written report of routine static [^18^F]FDG-PET/CT, but were blinded to all acquired dynamic PET data.

### FDG-PET/CT Scanning, Reconstruction, and Analysis

Whole-body dynamic PET images were obtained using a standard PET/CT scanner (DMI 6-ring PET/CT, GE HealthCare) with an automated multibed multipass dynamic whole-body acquisition protocol (dPET). Patients underwent imaging before the start of any treatment. FDG was injected on the scanner table, and a single bed over the heart was acquired simultaneously for 10 min, followed by 11 whole-body dynamic frames (46 min, 5 beds, 50 sec/bed). This was followed by a static acquisition at 60 min postinjection (1.5 min/bed), according to our institutional standards. All acquisitions were carried out in accordance with pertinent guidelines; detailed parameters used at our institution have been previously published.^29,30^

Reconstructed datasets, along with an aortic input function (IF) obtained from the PET dataset, were used to generate MR_FDG_ image datasets based on standard Patlak graphical analysis (11 frames, full IF) (DynamicIQ Advantage Workstation, GE HealthCare).

MR_FDG_ datasets of the dynamic acquisition and SUV datasets of the static acquisition were analyzed. In case of pathologic FDG uptake, VOIs were placed in vessel walls and joints for quantitation. The target-to-background ratio (TBR) was defined as the ratio of measured uptake in VOIs of pathologic vessel walls or joints (maximum values), related to VOIs in blood pool (for GCA), or to VOIs in physiological muscles (for PMR) (mean values).

Image acquisition of our “healthy” control group followed the same protocol as described above. In this control group, TBR ratios were measured and calculated in the same way as in the study group, with the vessel VOI being placed in the aortic arch or proximal portion of the descending aorta, and the joint VOI being placed into the left shoulder joint.

All image evaluations were performed by 2 experienced, doubly board-certified nuclear medicine physicians and radiologists in consensus, with 16 and 13 years of experience. VOI placement was done by a single reader, and the correct position of each VOI in 3 dimensions was assured by the second reader.

### Statistical Analysis

Intraindividual differences of parameters in patients with vasculitis or PMR were assessed by the Wilcoxon signed-rank test. Differences between the subjects of the vasculitis/PMR group and the echinococcus control group were analyzed by Mann-Whitney *U* test. A *P*-value of <0.05 was considered statistically significant. All analyses were performed using IBM SPSS Statistics™ 29.0.0 (SPSS Inc., Chicago, IL, USA).

## RESULTS

### Patients and Clinical Parameters

The median age in our study cohort was 61.3 years (range: 18.7–81.2). The median injected FDG activity was 180.6 MBq (range: 98–295). Mean serum C-reactive protein (CRP) level was 23.9 mg/L (range: 0.6–107), and mean erythrocyte sedimentation rate 52.4 mm/h (range: 16–80). At the time of imaging, no patient received steroids or any disease-specific treatment.

### Comparison of Standard Static PET and dPET

**FIGURE 2:**
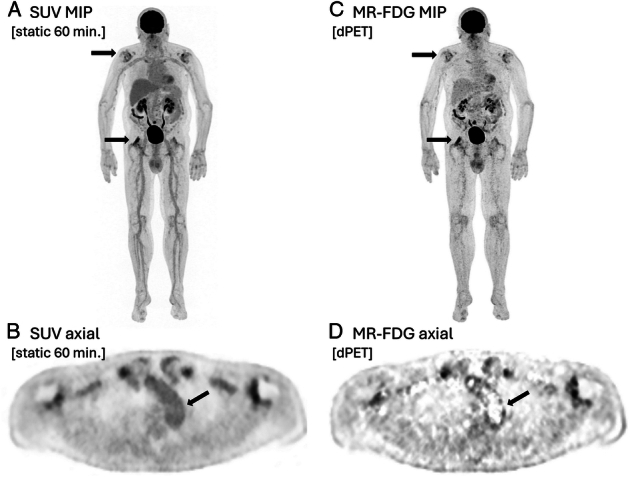
Patient with large-vessel vasculitis and polymyalgia rheumatica (PMR). **A** and **B**, Standard static SUV image dataset (TOF-BSREM, time-of-flight block sequential regularized expectation maximization, with a beta value of 450), acquired 60 min postinjection (1.5 min/bed). Greyscales-ranging from 0 to 8 (lower and upper limit, respectively). **C** and **D**, MR_FDG_ image dataset of multipass multibed dynamic acquisition (11 frames; blood pool + 46 min). Both reconstructions show increased FDG uptake in the large joints as sign of active PMR. In addition, MR_FDG_ images revealed increased FDG uptake in the aortic wall, represented by higher TBR of the aortic wall compared with static PET images, in line with the clinical diagnosis of large-vessel vasculitis.

**FIGURE 3:**
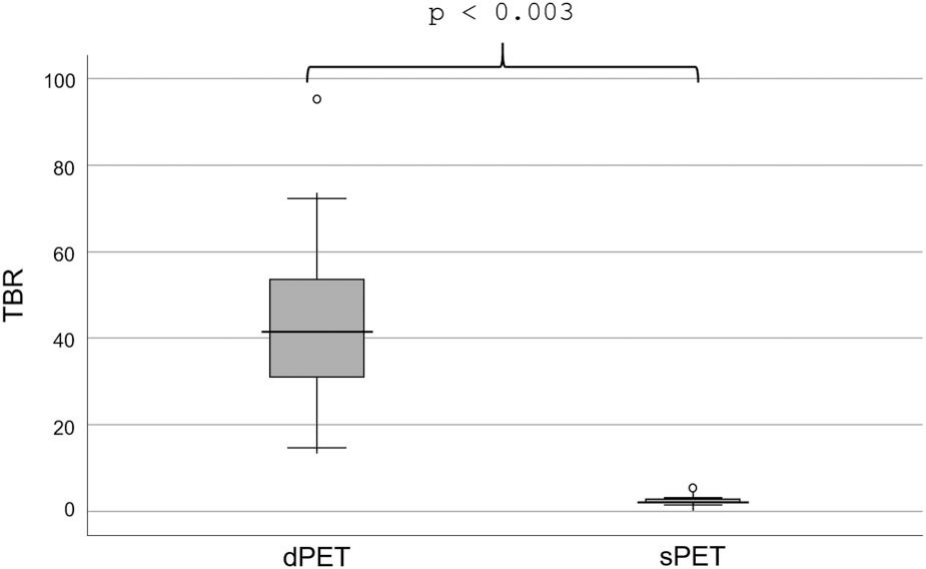
TBR in GCA. Boxplot of target-to-background ratio of dynamic whole-body PET (dPET; defined as MR_FDG___max_ vessel wall / MR_FDG___mean_ blood pool) and static PET (sPET; defined as SUV_max_ vessel wall / SUV_mean_ blood pool) in GCA patients.

Seventeen of 25 patients (68%) showed pathologic FDG uptake, of those, 5 (20%) were finally diagnosed with GCA alone, 6 (24%) with PMR alone, and another 6 (24%) with both conditions (Table 1). TBR of dPET (MR_FDG___max_ vessel wall/MR_FDG___mean_ blood pool) in GCA patients was 45.2 (range: 14.6–95.3), and TBR of static PET (SUV_max_ vessel wall/SUV_mean_ blood pool) was 2.5 (range: 1.4–5.4). TBR of dPET (MR_FDG___max_ joint/MR_FDG___mean_ muscle) in PMR was 10.1 (range: 5.3–23.8), and TBR of static PET (SUV_max_ joint/SUV_mean_ muscle) was 6.6 (range: 3.0–14.7). The difference in TBR between dPET and static PET was significant in both conditions (GCA *P*<0.003, PMR *P*<0.003) (Figs. 2, 3).

**TABLE 1:** Mean ± SD of the Measured Uptake in the VOIs.

	Giant Cell Arteritis (n=11)	Polymyalgia Rheumatica (n=12)	Control Group (n=11)
Vessel: MR_FDG___max_ vessel wall background: MR_FDG___mean_ blood pool	6.50 1.08 (3.15–12.39) 0.31±0.83 (0.03–0.95)		4.73±0.83* (1.50–8.69) 0.94±0.15 (0.13–1.79)
Vessel: SUV_max_ vessel wall background: SUV_mean_ blood pool	4.67±0.64 (2.84–9.90) 1.93±0.08 (1.61–2.47)		2.71±1.90* (2.23–4.29) 1.87±0.10 (1.40–2.51)
Joint: synovia MR_FDG___max_ joint background: MR_FDG___mean_ muscle		8.91±1.75 (1.07–22.33) 0.89±0.08 (0.20–1.32)	3.67±0.62 (1.06–7.07) 1.61±0.16 (0.93–2.75)
Joint: synovia SUV_max_ joint background: SUV_mean_ muscle		5.22±0.93 (2.27–12.65) 0.82±0.06 (0.57–1.16)	1.56±0.16 (0.83–2.53) 0.83±0.05 (0.62–1.11)

* measurement in healthy control group was done in the aortic arch.

### Comparison of TBR of PET-positive Patients to the Control Group

The difference in TBR between study group and control group was significant both for dPET as well as for static PET in both diseases (TBR of GCA vs. control group: dPET *P*<0.001 and static PET *P*<0.001; TBR of PMR vs. control group: dPET *P*<0.001 and static PET *P*<0.001).

### Comparison of TBR of PET-negative Patients to the Control Group

There was no significant difference in TBR between study patients without a final diagnosis of GCA or PMR and the control subjects, both with dPET as well as with static PET (dPET: *P*=0.075 and *P*=0.129 for vessels and synovia, respectively; static PET: *P*=0.968 and *P*=0.442 for vessels and synovia, respectively).

## DISCUSSION

To the best of our knowledge, our study is the first to assess the potential diagnostic value of dPET imaging in a collective of patients with vasculitis and PMR. We used a whole-body dynamic acquisition followed by standard Patlak analysis to subtract the unbound tracer from the image datasets. We demonstrated that the resulting MR_FDG_ datasets exhibited higher TBR compared with the standard static SUV datasets in both conditions. In addition, our study found significantly higher TBRs in patients with vasculitis or PMR, compared with subjects in the healthy control group, further supporting the validity of our approach. Our study confirms the potential of dPET imaging in distinguishing pathologic uptake from physiological background activity in rheumatic conditions.

[^18^F]FDG-PET/CT imaging is currently considered a standard imaging method in vasculitis and has made significant contributions to the diagnosis of PMR. Pooled meta-analyses report favorable diagnostic accuracies in large-vessel vasculitis, with sensitivities ranging from 76% to 88% and specificities between 71% and 95%.^11,31^ However, limitations may arise in detecting low disease activity and distinguishing inflammatory uptake from areas with high background signal.^20^ Standard static PET imaging can be particularly challenging in smaller vessels, where luminal activity may obscure vessel wall inflammation,^9^ as well as in traditionally difficult-to-image regions such as pulsating large vessels, and areas with variable or high background activity, such as muscles.

Whole-body dynamic PET acquisition addresses several limitations of standard PET imaging. By using a dynamic acquisition approach along with Patlak graphical analysis, it becomes possible to differentiate unbound tracer from the metabolized compound. This technique offers quantitative precision, which improves diagnostic accuracy compared with standard static imaging techniques. This methodological advancement helps reduce false positives, facilitates accurate quantification of metabolic activity, and enables more precise disease characterization.^7^ In our analyzed image datasets, we observed significantly improved TBR compared with standard static imaging. The elimination of the blood pool signal and enhanced joint capsule delineation are expected to result in more accurate disease characterization and potentially higher diagnostic accuracy than standard imaging techniques.^32^ The ability to precisely identify active inflammation is crucial in rheumatic diseases, and is expected to help monitor treatment response and define remission status.

Our study has limitations. We conducted consensus reading, since this is an exploratory analysis of the capabilities of dynamic PET imaging in patients with vasculitis or PMR. Future studies with larger sample sizes are needed to confirm our findings and focus on validating improved detectability. We used a multidisciplinary clinical consensus as the standard of reference, as histologic correlation was not feasible for all patients in our cohort. This included the clinically available standard PET images in individual patients, which might represent a form of selection bias. This composite standard of reference constitutes a major limitation of this study, as it includes PET data for classification, thereby introducing a selection bias and potentially limiting its validity. To partially address this, we compared our measurements and calculations to a healthy collective.

## CONCLUSIONS

WBD [^18^F]FDG-PET/CT yields significantly improved TBR compared with standard static imaging in patients with GCA and PMR. The elimination of the blood pool signal and enhanced joint capsule and vessel wall delineation are expected to lead to more accurate disease characterization and potentially higher diagnostic accuracy than standard static PET imaging.
